# Dietary acrylamide exposure and health risk assessment of pregnant women: A case study from Türkiye

**DOI:** 10.1002/fsn3.3828

**Published:** 2023-11-27

**Authors:** Hilal Pekmezci, Burhan Basaran

**Affiliations:** ^1^ Department of Elderly Care, Health Care Services Vocational School Recep Tayyip Erdogan University Rize Türkiye; ^2^ Department of Nutrition and Dietetics, Faculty of Health Sciences Recep Tayyip Erdogan University Rize Türkiye

**Keywords:** acrylamide, bread, carcinogenic risk, coffee, dietary exposure, French fries, hazard index

## Abstract

This study aimed to determine the acrylamide exposure of pregnant women resulting from the consumption of bread, coffee, and French fries and to evaluate it in terms of carcinogenic and non‐carcinogenic health risks. Retrospective 24‐h food consumption data of pregnant women (*n* = 487) was obtained using the Food Frequency Questionnaire (FFQ). Dietary acrylamide exposure was calculated according to a deterministic model, and the data were assessed by hazard index (HI) and carcinogenic risk (CR). The mean daily acrylamide exposure of pregnant women aged 18–30 and ≥31 years and in the 1st, 2nd, and 3rd trimesters was 31.4, 35.4, 38.7, 31.3, and 32.4 μg/day, respectively. The acrylamide exposure data were not significantly different among different age groups and pregnancy periods (*p* > .05). Dietary acrylamide exposure in pregnant women of different age groups and trimesters may cause significant and serious health problems in terms of carcinogenic risk. According to their level of contribution to average acrylamide exposure, the foods were ranked as follows: French fries> bread> coffee. There is a significant risk of cancer due to exposure to acrylamide from French fries and bread other than coffee. The findings suggest that pregnant women should avoid consuming French fries, bread, and coffee with high acrylamide levels for both their own health and their newborns' health.

## Graphical abstract

This study determined the acrylamide exposure of pregnant women resulting from the consumption of bread, coffee, and French fries and evaluated it in terms of carcinogenic and non‐carcinogenic health risks. Acrylamide exposure in pregnant women of different age groups and trimesters is not of concern in terms of non‐carcinogenic health risk (HI < 1) but may cause significant and serious health problems in terms of carcinogenic risk.

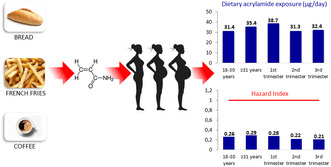

## 1 INTRODUCTION

Acrylamide (CAS No: 79‐06‐1), also known as acrylic amide, acrylamide, propenamide, and propenoic acid amide, is a colorless, odorless crystalline compound easily soluble in water and formulated as C_3_H_5_NO (National Center for Biotechnology Information, 2023). Acrylamide is a chemical agent that is widely used in many industries, such as in water and wastewater treatment plants, paper, textile, dye, and organic matter production, as well as in the construction of tunnels and sewers (International Programme on Chemical Safety, 2023). The presence of acrylamide in foods was first revealed by Swedish scientists in 2002 (Tareke et al., 2002).

The level of acrylamide in foods has been examined in many studies, including foods commonly consumed by different segments of society, such as bread, French fries, coffee, and biscuits, as well as ethnic and street foods (Andačić et al., 2020; Başaran & Turk, 2021; Cieslik et al., 2020; de Borba et al., 2023; Deribew & Woldegiorgis, 2021; Esposito et al., 2020; Lee et al., 2013; Mesias et al., 2022). The benchmark standards have been established for specific food types by Commission Regulation (EU) 2017/2158. The following amounts are considered the benchmarks for acrylamide in food: French fries (ready‐to‐eat): 500 μg/kg, soft bread: 50–100 μg/kg, roast coffee: 400 μg/kg, instant (soluble) coffee: 850 μg/kg, and coffee substitutes: 500–4000 μg/kg (Commission Regulation, 2017).

The Maillard reaction and the acrolein pathway are the two most widely accepted mechanisms of acrylamide formation in foods (Liska et al., 2016; Maan et al., 2022). The Maillard reaction is the reaction between free amino acids, such as asparagine, and reducing sugars, such as glucose and fructose, that occurs with heat treatment (Zyzak et al., 2003). It has been acknowledged that the Maillard reaction is the main formation mechanism of acrylamide in foods (Pesce et al., 2023). The acrolein pathway is that glycerol in oils heated above the smoke point forms acrolein; acrolein is then oxidized to acrylic acid, and acrylamide is formed in the presence of asparagine amino acids in the environment (Khorshidian et al., 2020).

Acrylamide, an extremely toxic compound, has mutagenic, neurotoxic, genotoxic, immunotoxic, and carcinogenic effects on animals (Amirshahrokhi, 2021; Dearfield et al., 1988; European Food Safety Authority, 2015). The International Agency for Research on Cancer (1994) identified acrylamide as a Group 2A probable carcinogen for humans. The European Commission classified acrylamide as a Category 2 reproductive toxicant and a Category 1B carcinogen and mutagen (European Commission, 2008). The European Chemical Agency (2021) included acrylamide in the List of Substances of Very High Concern. It has been reported that exposure to acrylamide may cause neurotoxic effects by irritating the eyes, skin, and respiratory tract (Hagmar et al., 2001; Kopanska et al., 2018). Several studies have shown that dietary acrylamide exposure is positively associated (Adani et al., 2020; Hogervost et al., 2008; Wilson et al., 2010), negatively associated (Atabati et al., 2020; Mucci et al., 2003), and not associated with various types of cancer (Filippini et al., 2022; Ikeda et al., 2021; Pelucchi et al., 2015). Gao et al. (2023) indicated that dietary acrylamide exposure may have an effect on the emotional status and neurobehavior of university students in China.

Pregnant women are vulnerable to heat treatment contaminants because of physical and chemical changes in their metabolism and bodies (Boyaci‐Gunduz, 2022; Fahim et al., 2023). Von Stedingk et al. (2011) stated that there was a strong correlation between acrylamide hemoglobin adducts measured in cord blood and those measured in maternal blood, and that acrylamide taken into the body during pregnancy could easily pass from the placenta to the fetus. It has been reported that dietary acrylamide exposure during pregnancy may cause neurotoxic effects in newborns (Dearfield et al., 1988) and may have negative effects on anthropometric characteristics such as low birth weight, birth length, and head circumference in children (Hogervorst et al., 2022; Pedersen et al., 2012; Zhan et al., 2020).

Diet is the primary source of acrylamide exposure for humans (Rifai & Saleh, 2020). The dietary exposure to acrylamide of individuals with different characteristics has been studied in many studies, and many of these studies reported that bread, coffee, and French fries contributed significantly to dietary acrylamide exposure (Abt et al., 2019; Costa et al., 2022; Fernández et al., 2022; Mojska et al., 2010; Mousavi Khaneghah et al., 2022). Although there are some studies (Brantsæter et al., 2008; Duarte‐Salles et al., 2013; Fernández et al., 2022; Kadawathagedara et al., 2016) on dietary exposure to acrylamide in pregnant women, current studies have not fully shed light on the explanation of the carcinogenic and non‐carcinogenic health risks of acrylamide exposure. The European Commission and Joint FAO/WHO Expert Committee on Food Additives suggest that more research is essential on dietary acrylamide exposure and health risk assessments (European Commission, 2019; Joint FAO/WHO Expert Committee on Food Additives, 2011). It has been reported that dietary acrylamide exposure in pregnant women should be reduced due to the potential risks of acrylamide, and further research should be conducted on this issue (Hogervorst et al., 2022; Pedersen et al., 2012). This study aimed to determine the dietary acrylamide exposure of pregnant women living in Türkiye with a variety of demographic characteristics by using actual consumption data on bread, coffee, and French fries and to evaluate it in terms of carcinogenic and non‐carcinogenic health risks.

## 2 MATERIALS AND METHODS

### 2.1 Ethical standards disclosure

This study was conducted under the “Principles of the Declaration of Helsinki”. With the decision letter dated January 24, 2020 and numbered 2020/04, written permission was granted from the ethics committee of the university where the study was conducted. In addition, institutional permission dated December 13, 2019 and numbered 64247179–799 was obtained from the Provincial Health Directorate. Since the use of a human phenomenon in the research requires the protection of individual rights, the “informed consent” condition was fulfilled as an ethical principle, and volunteering was taken as a basis. The idea of “respect for human dignity” was also considered in the study, and the “confidentiality principle” was followed by ensuring that information about participants would not be shared with anyone else.

### 2.2 Study population and data collection

No sample selection was made in the study. Between January 25, 2020 and July 30, 2022, the study population consisted of pregnant women who applied to two public hospitals and one private hospital in Türkiye for treatment, who voluntarily agreed to take part in the study, and who could understand the statements in the questionnaire. Face‐to‐face interviews were used to collect the data.

### 2.3 Dietary questionnaires

In this study, the retrospective 24‐h recall method was preferred to determine the pregnant women's consumption characteristics of French fries, bread, and coffee. In the first part of the food frequency questionnaire (FFQ), there are explanations about the purpose of the study, necessary ethical and administrative permission documents, names, contact information, and institutional information of the researchers, as well as questions to determine the age, educational status, number of children, gestational week, and chronic disease status of pregnant women. In the second part of the FFQ, questions were asked and recorded to determine whether pregnant women consumed bread, French fries, and coffee from waking up in the morning to going to bed, and thus to determine the amount of consumption.

### 2.4 Samples

The acrylamide levels in bread were taken from the most recent research on traditional and industrial bread sold locally and nationally in Türkiye (Başaran, 2022). In this study, the acrylamide levels of a total of 60 loaves of bread, including multi‐grain bread, whole‐meal bread, whole wheat bread, rye bread, and white bread, were determined (Table 1).

**TABLE 1:** Acrylamide levels in bread, coffee, and French fries (Başaran et al., 2020, 2022; Başaran & Turk, 2021).

Bread types	Mean (μg/kg)	French fries	Mean (μg/kg)	Coffee	Mean (μg/1 cup)
Multi‐grain bread	79.2	Sunflower oil	1037	3‐in‐1^a^	9.6
Wholemeal bread	83.5	Olive oil	1006	2‐in‐1 (sugar free)^a^	9.1
Whole wheat bread	76.8	Corn oil	1144	Latte^a^	6.8
Rye bread	82.6	Hazelnut oil	966	Cappuccino^a^	8.8
White bread	87.4			Americano^a^	3.9
				Filter coffee^a^	3.3
				Turkish coffee^b^	1.3
				Dibek coffee^b^	1.7

^a^ 1 cup = 200 mL.

^b^ 1 cup = 80 mL.

The acrylamide level of French fries was taken from the most recent survey based on the way people in Türkiye prepare them (Başaran & Turk, 2021). In this study, potatoes were fried 8 times consecutively in sunflower oil, olive oil, corn oil, and hazelnut oil, and the effects of oil type and consecutive use of oil in frying on acrylamide levels were examined (Table 1). In the study, the types of oil used by pregnant women in French fries and the sequential use of oil were neglected, and the acrylamide level of French fries was determined by taking the average of all the data obtained by frying 4 different types of oil for 8 consecutive times (1039 μg/kg).

The acrylamide level of coffee was taken from the most comprehensive and up‐to‐date research on packaged and ready‐to‐drink coffee sold locally and nationally in Türkiye (Başaran et al., 2020). In this study, the acrylamide levels were determined in a total of 41 coffee samples, including 22 samples of instant coffee, 7 samples of traditional Turkish coffee, and 12 samples of ready‐to‐drink (brewed) coffee. Yet, only the types of coffee consumed by pregnant women during the retrospective 24‐h period were included in this study (Table 1).

### 2.5 Health risk assessment

The acrylamide exposure of pregnant women was calculated using the following formula:

$$\mathrm{EDI}=\frac{F\times C\;}{\mathrm{bw}}$$

EDI refers to the estimated daily acrylamide intake (μg/kg bw/day), *F* is the amount of food consumed (g–mL day^−1^), *C* is the acrylamide concentration in the food (μg/kg–mL^−1^), and bw is the body weight (kg).

The portion amounts of each food group were determined as follows, using the book called “the Food and Nutrition Photo Catalog—Measurements and Quantities” by Rakıcıoğlu et al. (2012): For bread, 1 portion = 300 g, 1/2 portion = 150 g, and 1/4 portion = 75 g; for French fries, 1 portion = 90 g, 1/2 portion = 45 g, and 1/4 portion = 25 g; for Turkish and Dibek coffee, 1 cup = 80 mL; for all other coffee, 1 cup = 200 mL.

The target hazard quotient (THQ) describes the non‐carcinogenic health risk posed by exposure to the respective toxic compound. The THQ is defined as the ratio of exposure to the toxic compound to the reference dose, which is the highest level at which no adverse health effects are expected. The hazard index (HI) is the sum of the individual THQs of the elements assessed for each food type. The HI assumes that the consumption of a particular food type would result in simultaneous exposure to several potentially toxic compounds. In this case, the cumulative effect of the toxic compound may cause adverse health effects (Antoine et al., 2017; Basaran, 2022). While HI < 1 means there is no concern about a health risk, HI ≥ 1 indicates a potential health problem that is not carcinogenic (United States Environmental Protection Agency, 1989). HI was calculated according to the formula as follows:

$$\mathrm{HI}=\sum \left({\mathrm{THQ}}_{\text{bread}}=\frac{\mathrm{EDI}}{\mathrm{RfD}}\right)+\left({\mathrm{THQ}}_{\text{coffee}}=\frac{\mathrm{EDI}}{\mathrm{RfD}}\right)+\left({\mathrm{THQ}}_{\text{French fries}}=\frac{\mathrm{EDI}}{\mathrm{RfD}}\right)$$

EDI stands for the chronic daily intake (μg/kg bw/day), and RfD (reference dose) determined for acrylamide is 2 × 10^−3^ mg/kg bw/day (United States Environmental Protection Agency, 2010).

The carcinogenic risk (CR) value determines the cancer risk that a population may be exposed to over the course of their lifetime due to a carcinogenic or potentially carcinogenic substance (United States Environmental Protection Agency, 2021). CR < 1.00 × 10^−6^ is regarded as safe and insignificant; 1.00 × 10^−4^ < CR < 1.00 × 10^−6^ is considered a probable and significant risk; 1.00 × 10^−4^ < CR is considered a serious health risk.

CR=EDI×OSF

EDI stands for the chronic daily intake (μg/kg bw/day), and OSF refers to the oral slope factor. OSF for acrylamide is determined to be 5 × 10^−1^ mg/kg/day (United States Environmental Protection Agency, 2010).

### 2.6 Statistical analysis

The analysis was ended by transferring the study data to the IBM SPSS Statistics 26 program. While evaluating the data, frequency distributions for categorical variables and descriptive statistics (mean ± SD, median) for numerical variables were given. The Kolmogorov–Smirnov normality test (*n* > 30) was applied to the data to decide on the analyses to be applied. It was observed that the values were not in line with the assumption of a normal distribution, so nonparametric tests were employed. The difference between two independent groups was analyzed by the Mann–Whitney *U* test, the difference between more than two independent groups was analyzed by the Kruskal–Wallis analysis, and the difference between more than two dependent groups was analyzed by the Friedman test. The Bonferroni test was used to assess the difference between the groups. Different letters in the same group show statistically significant differences (*p* < .05).

## 3 RESULTS AND DISCUSSION

### 3.1 Demographic characteristics of pregnant women

The study involved 487 pregnant women. The average body weight of pregnant women was 75.6 kg, their length was 163 cm, and their body mass index was 28.5 kg/m^2^. Approximately 71.0% of the participants were in the 18–30 age range, 9.90% were in the first trimester of their pregnancy (0–14 weeks), 32.6% were in the second trimester of their pregnancy (15–28 weeks), and 57.5% were in the third trimester of their pregnancy (29–40 weeks). The average amount of bread and French fries consumed by pregnant women aged ≥31 years was significantly higher than that of pregnant women aged 18–30 years (*p* < .05). The average amount of bread consumption of pregnant women in the 3rd trimester was significantly higher than that of pregnant women in the 1st and 2nd trimesters; the average amount of bread consumption of pregnant women in the 2nd trimester was significantly higher than that of pregnant women in the 1st trimester; and the average amount of French fries consumption of pregnant women in the 1st trimester was significantly higher than that of pregnant women in the 2nd and 3rd trimesters (*p* < .05). The average body mass index of 52.2% of pregnant women indicated that they were overweight. It was observed that as body mass index increased, coffee consumption decreased, while there was no change in bread and French fry consumption. 43.3% of the participants were high school graduates, and it was seen that as the education level increased, the consumption of bread and French fries decreased, but the consumption of coffee increased. Seventy percent of pregnant women had 1–2 children. It was observed that as the number of children increased, the consumption of bread and French fries increased, but the consumption of coffee decreased. Seventy‐four percent of pregnant women did not have any known chronic diseases. It was observed that pregnant women with chronic diseases consumed more bread and French fries than women without chronic diseases. None of the pregnant women included in the study were smokers (Table 2).

**TABLE 2:** Demographic characteristics of pregnant women.

Demographic characteristics	Number of participants (*n* = 487)	Average body weight (kg)	Average height (cm)	Average bread consumption (g)	Average coffee consumption (mL)	Average french fries consumption (g)
Age ranges						
18–30	344	74.8 ± 12.4	1.63 ± 0.10	122^b^	29.0^ns^	20.0^b^
≥31	143	78.7 ± 10.3	1.63 ± 0.09	140^a^	32.0^ns^	23.0^a^
Gestational age						
1. trimester^a^	48	69.1 ± 12.0	1.65 ± 0.11	110^c^	28.5^ns^	24.0^a^
2. trimester^b^	159	72.7 ± 19.8	1.63 ± 0.05	120^b^	29.0^ns^	20.0^b^
3. trimester^c^	280	79.0 ± 12.2	1.63 ± 0.08	134^a^	31.0^ns^	20.0^b^
Average BMI index (kg/m^2^)						
Normal (18.5–24.9)	89	62.4 ± 6.49	1.64 ± 0.07	130	30.0	20.0
Overweight (25–29.9)	254	73.3 ± 7.41	1.63 ± 0.07	130	28.0	20.0
Obese (>30)	144	86.7 ± 12.4	1.62 ± 0.05	130	26.0	20.0
Education level						
Primary education	88	75.3 ± 5.66	1.61 ± 0.07	148	30.0	32.0
High school	211	75.0 ± 9.50	1.63 ± 0.06	127	26.0	19.0
Associate degree	97	75.3 ± 10.1	1.64 ± 0.08	127	24.5	17.0
Undergraduate degree+	91	76.0 ± 12.0	1.65 ± 0.07	109	48.0	16.0
Number of children						
0	68	73.3 ± 8.50	1.62 ± 0.05	123	41.0	15.0
1–2	339	75.2 ± 11.4	1.62 ± 0.08	127	27.5	21.0
3+	80	75.2 ± 10.8	1.63 ± 0.07	131	31.0	24.0
Chronic disease status						
Yes	127	77.2 ± 9.90	1.63 ± 0.08	133	24.0	27.0
No	360	74.6 ± 10.5	1.63 ± 0.07	125	32.0	18.0

*Note*: Different letters in the same group indicate statistically significant differences.

Abbreviation: ns, not significant.

^a^ 1. trimester = 0–14 weeks.

^b^ 2. trimester = 15–28 weeks.

^c^ 3. trimester = 29–40 weeks.

### 3.2 Dietary acrylamide exposure

The type and amount of French fries, coffee, and bread consumed by each pregnant woman in the 24‐h retrospective period were transferred to the tables in such a way that they stood in the same line as the same individual, and dietary acrylamide exposure was calculated (Table 3).

**TABLE 3:** Examination of the difference between age and gestational age groups according to dietary acrylamide exposure values.

EDI (μg/kg bw/day)	Age ranges		Gestational age		
	18–30	≥31	1. Trimester^a^	2. Trimester^b^	3. Trimester^c^
	Median (min.–max.)	Median (min.–max.)	Median (min.–max.)	Median (min.–max.)	Median (min.–max.)
Bread					
By body weight	0.11 (0.00–2.27)^ns^	0.14 (0.00–0.49)^ns^	0.11 (0.00–1.75)^ns^	0.11 (0.00–0.38)^ns^	0.14 (0.00–2.27)^ns^
By body mass index	0.28 (0.00–5.95)^ns^	0.36 (0.00–1.29)^ns^	0.27 (0.00–4.20)^a^	0.27 (0.00–0.98)^ns^	0.37 (0.00–5.95)^ns^
Coffee					
By body weight	0.00 (0.00–0.30)^ns^	0.00 (0.00–0.17)^ns^	0.00 (0.00–0.17)^ns^	0.00 (0.00–0.30)^ns^	0.00 (0.00–0.17)^ns^
By body mass index	0.00 (0.00–0.82)^ns^	0.00 (0.00–0.41)^ns^	0.00 (0.00–0.40)^ns^	0.00 (0.00–0.82)^ns^	0.00 (0.00–0.43)^ns^
French fries					
By body weight	0.00 (0.00–1.73)^ns^	0.00 (0.00–1.95)^ns^	0.00 (0.00–1.39)^ns^	0.00 (0.00–1.73)^ns^	0.00 (0.00–1.95)^ns^
By body mass index	0.00 (0.00–5.00)^ns^	0.00 (0.00–4.99)^ns^	0.00 (0.00–3.86)^ns^	0.00 (0.00–5.00)^ns^	0.00 (0.00–4.99)^ns^
Total EDI					
By body weight	0.19 (0.00–2.27)^ns^	0.20 (0.00–2.10)^ns^	0.37 (0.00–1.75)^ns^	0.18 (0.00–1.80)^ns^	0.19 (0.00–2.27)^ns^
By body mass index	0.52 (0.00–5.95)^ns^	0.50 (0.00–5.48)^ns^	0.98 (0.00–4.20)^ns^	0.50 (0.00–5.20)^ns^	0.49 (0.00–5.95)^ns^

Abbreviations: EDI, estimated daily acrylamide intake; ns, not significant.

^a^ 1. trimester = 0–14 weeks.

^b^ 2. trimester = 15–28 weeks.

^c^ 3. trimester = 29–40 weeks.

**FIGURE 1:**
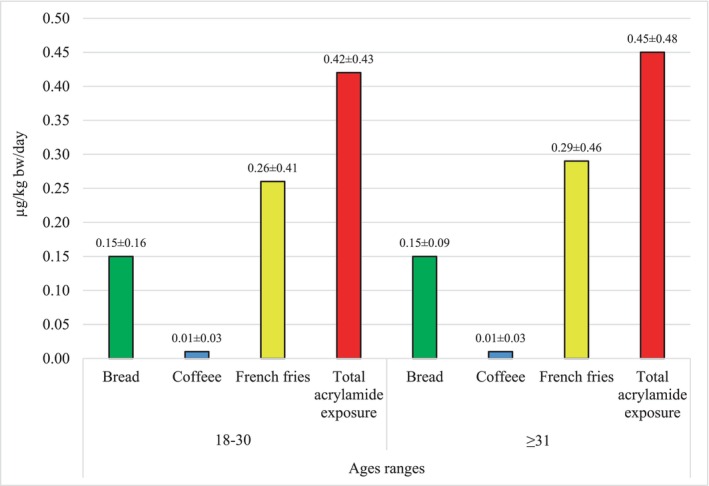
Average acrylamide exposure of pregnant women by age groups (by body weight).

The mean total acrylamide exposure from bread, French fries, and coffee consumption was 0.42 ± 0.43 and 0.45 ± 0.48 μg/kg bw/day for pregnant women aged 18–30 and ≥31, respectively, based on body weight. The mean total acrylamide exposure from bread, French fries, and coffee consumption was 1.12 ± 1.15 and 1.19 ± 1.25 μg/kg bw/day for pregnant women aged 18–30 and ≥31, respectively, based on body mass index. Pregnant women aged ≥31 may be expected to have higher levels of acrylamide exposure because their consumption of bread, coffee, and French fries is higher than that of pregnant women aged 18–30. There was no significant difference between acrylamide exposure from bread, coffee, and French fries consumption and the total acrylamide exposure levels (median) of pregnant women in different age groups according to body weight and body mass index (*p* > .05) (Table 3). The average acrylamide exposure levels of pregnant women of different age ranges resulting from consumption of only bread, French fries, and coffee are shown in Figure 1. According to average acrylamide exposure levels in both age groups, foods are listed as French fries > bread > coffee.

**FIGURE 2:**
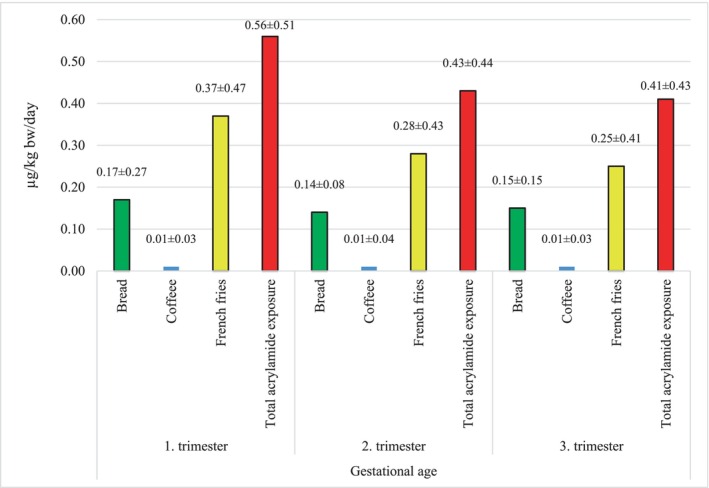
Average acrylamide exposure of pregnant women by trimester (by body weight).

The mean total acrylamide exposure from bread, French fries, and coffee consumption was calculated as 0.56 ± 0.51, 0.43 ± 0.44, and 0.41 ± 0.43 μg/kg bw/day for pregnant women in the 1st, 2nd, and 3rd trimesters, respectively, based on body weight. The mean total acrylamide exposure from bread, French fries, and coffee consumption was calculated as 1.48 ± 1.37, 1.16 ± 1.18, and 1.08 ± 1.14 μg/kg bw/day, respectively, based on body weight. Pregnant women in the 1st trimester had lower body weight than pregnant women in other trimesters and consumed higher amounts of French fries, which have higher acrylamide levels than other foods, which explains the high acrylamide exposure. Although pregnant women in the 3rd trimester consumed more bread and coffee than those in the 2nd trimester, the lower body weight of pregnant women in the 2nd trimester is the main reason for the difference in exposure. There was no significant difference between acrylamide exposure from each food consumption and the total acrylamide exposure levels (median) of pregnant women in different trimesters according to body weight and body mass index (*p* > .05) (Table 3). The average acrylamide exposure levels of pregnant women in different trimesters, resulting only from consumption of bread, French fries, and coffee, are shown in Figure 2. According to average acrylamide exposure levels in different trimester periods, foods are listed as French fries > bread > coffee.

The Joint FAO/WHO Expert Committee on Food Additives estimated average acrylamide exposure for the general population (1 μg/kg bw per day) and for consumers in the high percentile (4 μg/kg bw per day). The committee determined the No Observed Adverse Effect Level (NOAEL) for acrylamide as 200 μg/kg bw/day (namely, non‐carcinogenic morphological changes in nerves, detected by electron microscopy, in rats). Therefore, in this study, the mean acrylamide levels calculated for both age groups and pregnant women in different trimesters were well below the acrylamide set by the Joint FAO/WHO Expert Committee on Food Additives (2011). Brantsæter et al. (2008) found that pregnant women's dietary acrylamide exposure (median) was 0.48 μg/kg bw/day, Chan‐Hon‐Tong et al. (2013) found that women's mean whole‐food acrylamide exposure (LB/UB) was 0.40–0.41 and 0.28–0.29 μg/kg bw/day, Duarte‐Salles et al. (2013) estimated the mean acrylamide exposure in pregnant women as 0.4 ± 0.2 μg/kg bw/day in a study conducted in Norway using the data from the Mother and Child Cohort Study (MoBa). Kadawathagedara et al. (2016) calculated dietary acrylamide exposure in pregnant women (French EDEN mother–child cohort study) as 0.33 ± 0.25 μg/kg bw/day (23.8 ± 17.3 μg/day). Fernández et al. (2022) reported that 100% of lactating women in Spain were exposed to acrylamide (mean EDI = 1.2–1.9 μg/kg bw/day). In a meta‐analysis study, Hogervorst et al. (2022), who calculated the average dietary acrylamide exposure in pregnant women as 21.9 μg/day, reported that there may be differences in acrylamide exposure between trimesters.

The findings on acrylamide exposure in this study are consistent with those of Brantsæter et al. (2008) and Duarte‐Salles et al. (2013). On the other hand, the study obtained higher acrylamide exposure levels than those of Chan‐Hon‐Tong et al. (2013), Kadawathagedara et al. (2016), and Hogervorst et al. (2022). The main reason for the differences between the studies is undoubtedly the different levels of acrylamide in foods and the diverse dietary habits according to geography and culture. Women follow a special dietary program during pregnancy, both to eat healthily and to avoid gaining too much weight. Therefore, this means that women are exposed to less acrylamide before pregnancy than in their normal lives. Indeed, as in the studies by Chan‐Hon‐Tong et al. (2013) and Fernández et al. (2022), increases or decreases in dietary acrylamide exposure could be expected since pre‐ and postpartum diets are different from each other.

### 3.3 Health risk assessment

CR, THQ, and HI values were determined with reference to the mean acrylamide levels calculated according to the body weight of pregnant women (Tables 4 and 5).

**TABLE 4:** Examining the difference between age and gestational age groups according to non‐carcinogenic risk values.^d^

Food groups	Age ranges		Gestational age		
	18–30	≥31	1. trimester^a^	2. trimester^b^	3. trimester^c^
	Median (min.–max.)	Median (min.–max.)	Median (min.–max.)	Median (min.–max.)	Median (min.–max.)
Bread (THQ)	0.05 (0.00–1.13)^ns^	0.07 (0.00–0.24)^ns^	0.05 (0.00–0.87)^ns^	0.05 (0.00–0.19)^ns^	0.07 (0.00–1.13)^ns^
Mean	0.07 ± 0.08	0.08 ± 0.05	0.09 ± 0.14	0.07 ± 0.04	0.08 ± 0.08
Coffee (THQ)	0.00 (0.00–0.15)^ns^	0.00 (0.00–0.08)^ns^	0.00 (0.00–0.08)^ns^	0.00 (0.00–0.15)^ns^	0.00 (0.00–0.08)^ns^
Mean	0.006 ± 0.026	0.006 ± 0.015	0.007 ± 0.017	0.006 ± 0.018	0.006 ± 0.014
French fries (THQ)	0.00 (0.00–0.87)^ns^	0.00 (0.00–0.97)^ns^	0.00 (0.00–0.70)^ns^	0.00 (0.00–0.87)^ns^	0.00 (0.00–0.97)^ns^
Mean	0.13 ± 0.20	0.15 ± 0.23	0.19 ± 0.23	0.14 ± 0.21	0.12 ± 0.21
Hazard index (HI)^e^	0.10 (0.00–1.13)^ns^	0.10 (0.00–1.05)^ns^	0.19 (0.00–0.87)^ns^	0.09 (0.00–0.90)^ns^	0.10 (0.00–1.13)^ns^
Mean	0.26 ± 0.22	0.29 ± 0.24	0.28 ± 0.25	0.22 ± 0.22	0.21 ± 0.22

Abbreviation: ns, not significant.

^a^ 1. trimester = 0–14 weeks.

^b^ 2. trimester = 15–28 weeks.

^c^ 3. trimester = 29–40 weeks.

^d^ Acrylamide exposure values were taken from body weight values.

^e^ Hazard index (HI) is a non‐carcinogenic potential health index and is calculated by summing target hazard quotient (THQ) values.

**TABLE 5:** Examination of the difference between age and gestational age groups according to carcinogenic risk values.

Food groups	Age ranges		Gestational age		
	18–30	≥31	1. trimester^a^	2. trimester^b^	3. trimester^c^
	Median (min.–max.)	Median (min.–max.)	Median (min.–max.)	Median (min.–max.)	Median (min.–max.)
Bread	5.46 × 10^−5^ (0.00–1.13 × 10^−3^)^ns^	6.95 × 10^−5^ (0.00–2.43 × 10^−4^)^ns^	5.42 × 10^−5^ (0.00–8.74 × 10^−4^)^ns^	5.28 × 10^−5^ (0.00–1.89 × 10^−4^)^ns^	6.95 × 10^−5^ (0.00–1.13 × 10^−3^)^ns^
Mean	7.38 × 10^−5^ ± 8.24 × 10^−5^	7.54 × 10^−5^ ± 4.67 × 10^−5^	8.64 × 10^−5^ ± 1.37 × 10^−4^	6.92 × 10^−5^ ± 3.75 × 10^−5^	7.54 × 10^−5^ ± 7.72 × 10^−5^
Coffee	0.00 (0.00–1.52 × 10^−4^)^ns^	0.00 (0.00–8.28 × 10^−4^)^ns^	0.00 (0.00–8.43 × 10^−5^)^ns^	0.00 (0.00–1.52 × 10^−4^)^ns^	0.00 (0.00–8.28 × 10^−5^)^ns^
Mean	5.80 × 10^−6^ ± 1.65 × 10^−5^	5.80 × 10^−6^ ± 1.45 × 10^−5^	6.60 × 10^−6^ ± 1.70 × 10^−5^	6.00 × 10^−6^ ± 1.82 × 10^−5^	5.60 × 10^−6^ ± 1.43 × 10^−5^
French fries	0.00 (0.00–8.66 × 10^−4^)^ns^	0.00 (0.00–9.74 × 10^−4^)^ns^	0.00 (0.00–6.96 × 10^−4^)^ns^	0.00 (0.00–8.66 × 10^−4^)^ns^	0.00 (0.00–9.74 × 10^−4^)^ns^
Mean	1.31 × 10^−4^ ± 2.04 × 10^−4^	1.45 × 10^−4^ ± 2.29 × 10^−4^	1.85 × 10^−4^ ± 2.33 × 10^−4^	1.41 × 10^−4^ ± 2.14 × 10^−4^	1.24 × 10^−4^ ± 2.05 × 10^−4^
CR	9.56 × 10^−5^ (0.00–1.13 × 10^−3^)^ns^	9.95 × 10^−5^ (0.00–1.05 × 10^−3^)^ns^	1.85 × 10^−4^ (0.00–8.74 × 10^−4^)^ns^	8.99 × 10^−5^ (0.00–8.99 × 10^−4^)^ns^	9.63 × 10^−5^ (0.00–1.13 × 10^−3^)^ns^
Mean	2.10 × 10^−4^ ± 2.15 × 10^−4^	2.26 × 10^−4^ ± 2.38 × 10^−4^	2.78 × 10^−4^ ± 2.55 × 10^−5^	2.16 × 10^−4^ ± 2.22 × 10^−5^	2.05 × 10^−4^ ± 2.16 × 10^−4^

Abbreviations: CR, carcinogenic risk; ns, not significant.

^a^ 1. trimester = 0–14 weeks.

^b^ 2. trimester = 15–28 weeks.

^c^ 3. trimester = 29–40 weeks.

When the data in Table 4 were analyzed, it was seen that the mean THQ values of pregnant women aged 18–30 and 31> were 0.07 ± 0.08 and 0.08 ± 0.05 (bread), 0.006 ± 0.026 and 0.006 ± 0.015 (coffee), 0.13 ± 0.20 and 0.15 ± 0.23 (French fries), respectively. The mean THQ values of pregnant women in the 1st, 2nd, and 3rd trimesters were 0.09 ± 0.14, 0.07 ± 0.04, and 0.08 ± 0.08 (bread), 0.007 ± 0.017, 0.006 ± 0.018, and 0.006 ± 0.014 (coffee), 0.19 ± 0.23, 0.14 ± 0.21, and 0.12 ± 0.21 (French fries), respectively. The mean HI values (total THQ) were 0.26 ± 0.22 and 0.29 ± 0.24 in pregnant women aged 18–30 and ≥31, respectively, and 0.28 ± 0.25, 0.22 ± 0.22, and 0.21 ± 0.22 in pregnant women in the 1st, 2nd, and 3rd trimesters, respectively. While HI values increased in pregnant women, depending on the increase in age, they decreased during the trimesters. There was no significant difference between age groups and trimesters according to the mean THQ resulting from each food consumption and the total THQ (HI) values (median) (*p* > .05). The THQ and HI values for each food consumed by pregnant women in different age groups and trimesters are noticeably much <1, which is the reference value determined by the United States Environmental Protection Agency (1989). Therefore, it is very difficult to talk about a potential hazard in terms of non‐carcinogenic health risks.

When the data in Table 5 were examined, it was seen that the mean total CR values of acrylamide exposure from bread, coffee, and French fries consumption were determined as 2.10 × 10^−4^ and 2.26 × 10^−4^ for pregnant women aged 18–30 and 31>, respectively, and 2.78 × 10^−4^, 2.16 × 10^−4^, and 2.05 × 10^−4^ in pregnant women in the 1st, 2nd, and 3rd trimesters, respectively. There was no significant difference between age groups and trimesters according to the CR values resulting from each food consumption and the mean total CR values (median) (*p* > .05). CR < 1.00 × 10^−6^ is regarded as safe and insignificant; 1.00 × 10^−4^ < CR <1.00 × 10^−6^ is considered a probable and significant risk; 1.00 × 10^−4^ < CR is considered a serious health risk. Since the mean total CR values calculated in pregnant women of different ages and trimesters are >1.00 × 10^−4^, the findings refer to a very serious carcinogenic health problem.

Very few studies in the literature have examined the carcinogenic and non‐carcinogenic health risks caused by dietary acrylamide exposure in pregnant women. Chan‐Hon‐Tong et al. (2013) reported that dietary acrylamide exposure in pregnant women in the 3rd trimester carried no carcinogenic risk (margin of exposure approach), while Fernández et al. (2022) reported that dietary acrylamide exposure in lactating women in Spain posed significant carcinogenic (margin of exposure approach) and non‐carcinogenic risks.

**FIGURE 3:**
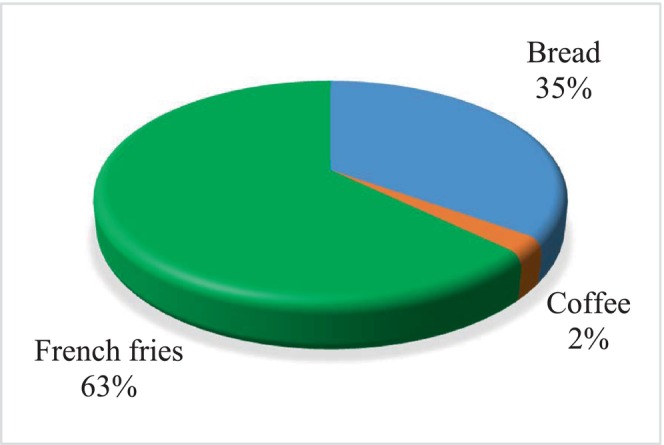
Contribution levels of food groups to the mean acrylamide level according to body weight.

Since the acrylamide level of each type of food is different and these foods are consumed in different amounts, the contribution levels of each food to the acrylamide exposure of pregnant women are also different. Pregnant women consume less French fries than bread, based on their average consumption in a retrospective 24‐h period. However, since the acrylamide level of French fries is much higher than that of other foods, their contribution to acrylamide exposure was higher despite the low consumption amount (Figure 3). Therefore, within the scope of the study, the carcinogenic and non‐carcinogenic risk values of acrylamide exposure resulting from the consumption of bread, coffee, and French fries were also calculated (Table 6). The mean acrylamide exposure levels of pregnant women with different demographic characteristics resulting from consumption of bread, coffee, and French fries alone were determined as 0.15 ± 0.15, 0.01 ± 0.03, and 0.27 ± 0.42 μg/kg bw/day, respectively, according to body weight, and 0.39 ± 0.38, 0.03 ± 0.09, and 0.72 ± 1.12 μg/kg bw/day, respectively, according to body mass index. According to body weight, the acrylamide exposure (median) caused by the consumption of bread was found to be significantly higher than the exposure to French fries and coffee, and the acrylamide exposure (median) caused by the consumption of French fries was found to be significantly higher than the acrylamide exposure due to coffee consumption (*p* < .05).

**TABLE 6:** Examining the difference between food groups according to EDI, THQ, and CR values.

Food groups	EDI by body weight (μg/kg bw/day)	EDI by body mass index (μg/kg bw/day)	THQ	CR
	Median (min.–max.)	Median (min.–max.)	Median (min.–max.)	Median (min.–max.)
Bread	0.13 (0.00–2.27)^a^	0.34 (0.00–5.95)^a^	0.06 (0.00–1.13)^a^	6.38 × 10^−5^ (0.00–1.13 × 10^−3^)^a^
Mean	0.15 ± 0.15	0.39 ± 0.38	0.07 ± 0.07	7.43 × 10^−5^
Coffee	0.00 (0.00–0.30)^c^	0.00 (0.00–0.82)^c^	0.00 (0.00–0.15)^c^	0.00 (0.00–1.52 × 10^−4^)^c^
Mean	0.01 ± 0.03	0.03 ± 0.09	0.006 ± 0.016	5.80 × 10^−6^
French fries	0.00 (0.00–1.95)^b^	0.00 (0.00–5.00)^b^	0.00 (0.00–0.97)^b^	0.00 (0.00–9.74 × 10^−4^)^b^
Mean	0.27 ± 0.42	0.72 ± 1.12	0.14 ± 0.21	1.35 × 10^−4^

*Note*: Different letters in the same group indicate statistically significant differences.

Abbreviations: CR, carcinogenic risk; EDI, estimated daily acrylamide intake; THQ, target hazard quotient.

^a^ 1. Trimester = 0–14 weeks.

^b^ 2. Trimester = 15–28 weeks.

^c^ 3. Trimester = 29–40 weeks.

The mean THQ values of bread, coffee, and French fries were calculated as 0.07 ± 0.07, 0.006 ± 0.016, and 0.14 ± 0.21, respectively. Since THQ < 1, the values show that the consumption of any food will not pose a potential danger. The mean CR values for bread, coffee, and French fries were determined to be 7.43 × 10^−5^, 5.80 × 10^−6^, and 1.35 × 10^−4^, respectively. According to the CR values, the consumption of French fries leads to a serious health risk, the consumption of bread causes a potential and significant health risk, and the consumption of coffee does not pose any health risk. THQ and CR values (median) resulting from bread consumption were found to be significantly higher than those resulting from French fries and coffee consumption, and THQ, and CR values (median) resulting from French fries consumption were significantly higher (*p* < .05). The majority of pregnant women consume bread frequently in a retrospective 24‐h period. However, the same individuals consumed French fries and coffee less frequently during the same period. That is why the median value of bread was higher than that of French fries and coffee. When the median values of the relevant foods were taken, the exposure, THQ, and CR values resulting from bread consumption were higher. Therefore, acrylamide exposure from bread consumption affected a greater number of pregnant women.

Türkiye ranks first in bread consumption in the world, which explains the significant contribution of bread to acrylamide exposure. In addition, Turkish coffee, which is registered as an intangible cultural heritage, is frequently consumed by society. However, the small volume (80 mL) of the cup used in the traditional consumption of Turkish coffee is thought to limit acrylamide exposure. In this study, the findings regarding the foods that contribute to the exposure of pregnant women to acrylamide are consistent with the literature. Brantsæter et al. (2008) reported that the primary sources of total acrylamide exposure in pregnant women were potato chips, crisp breads, biscuits, breakfast cereals, and bakery products, while Chan‐Hon‐Tong et al. (2013) stated that the foods that contributed most to dietary acrylamide exposure in pregnant women in the 3rd trimester were potatoes and related products, biscuits, coffee, and bread. Pedersen et al. (2012) linked maternal consumption of acrylamide‐rich foods, such as fried potatoes, with acrylamide detected in cord blood and low birth weight. Kadawathagedara et al. (2016) found a high correlation between dietary acrylamide exposure and French fries, a low correlation between tea and coffee, and the lowest correlation between bread and biscuits in pregnant women. Zhan et al. (2020) reported that the primary food sources of acrylamide exposure in pregnant women were French fries, chips, bread, and coffee. Fernández et al. (2022) reported that coffee, bakery products, and pre‐cooked products were strongly associated with high levels of acrylamide metabolites detected in urine in lactating women.

## 4 CONCLUSIONS

Acrylamide is a compound that is found at different levels in many foods frequently consumed in daily life, occurs naturally in foods through heat treatment, and is identified as possibly carcinogenic to humans. Acrylamide is also considered a major concern by many researchers and institutions due to its potential risks. In this study, the acrylamide exposure of pregnant women, which is a highly sensitive group, resulting from the consumption of bread, coffee, and French fries, which are considered rich in acrylamide formation and are frequently consumed, was evaluated in terms of health risks.

The mean acrylamide exposure of all pregnant women (*n* = 487) resulting from the consumption of bread, coffee, and French fries was calculated as 0.43 ± 0.44 μg/kg bw/day based on body weight and 1.14 ± 1.18 μg/kg bw/day based on body mass index. Since the mean HI of pregnant women (0.22 ± 0.22) was <1, there is no cause for concern in terms of non‐carcinogenic health risk. The mean CR value was 2.15 × 10^−4^ ± 2.18 × 10^−4^, showing a serious carcinogenic health risk. Among the food types included in the study, bread and French fries were also found to have high CR values. It is understood that the type and amount of food consumed are important components in terms of their effect on dietary acrylamide exposure and health risks. Therefore, businesses should reduce the level of acrylamide in foods, and individuals should be educated about risky foods in terms of acrylamide formation. This synergistic effect could lead to significant reductions in dietary acrylamide exposure. Further studies are needed to determine whether dietary acrylamide exposure leads to adverse health effects and mobilize industry and society.

## AUTHOR CONTRIBUTIONS

**Hilal Pekmezci:** Conceptualization (equal); data curation (equal); formal analysis (equal); funding acquisition (equal); investigation (equal); methodology (equal); project administration (equal); resources (equal); software (equal); supervision (equal); validation (equal); visualization (equal); writing – original draft (equal); writing – review and editing (equal). **Burhan Basaran:** Conceptualization (equal); data curation (equal); formal analysis (equal); funding acquisition (equal); investigation (equal); methodology (equal); project administration (equal); resources (equal); software (equal); supervision (equal); validation (equal); visualization (equal); writing – original draft (equal); writing – review and editing (equal).

## FUNDING INFORMATION

This research did not receive any specific grants from funding agencies in the public, commercial, or not‐for‐profit sectors.

## CONFLICT OF INTEREST STATEMENT

The authors declare that they have no competing interests.

## ETHICS STATEMENT

This study was carried out with the permission of the Recep Tayyip Erdogan University Ethics Committee (Report: 2020/04) and the Rize Provincial Health Directorate (Report: 64247179‐799).

## CONSENT TO PARTICIPATE

An informed consent form was signed by all participants before recruiting for the study.

## Data Availability

The datasets used and analyzed during the current study are available from the corresponding author on reasonable request.
